# Brain Metastases in Adults: A Five-Year Observational Study From King Abdulaziz Medical City

**DOI:** 10.7759/cureus.31197

**Published:** 2022-11-07

**Authors:** Jawahir O AlTamimi, Hadeel A AlJohani, Nada Naaman, Reshale A Johar, Tala A Allam, Dr. Ahmed I Lary

**Affiliations:** 1 College of Medicine, King Saud bin Abdulaziz for Health Sciences, Jeddah, SAU; 2 College of Medicine, King Saud Bin Abdulaziz for Health Sciences, Jeddah, SAU; 3 Department of Neurosurgery, King Abdulaziz Medical City, Jeddah, SAU

**Keywords:** epidemiology, breast cancer, lung cancer, metastases, brain metastases

## Abstract

Background

As a well-documented fact, metastatic brain tumors are the most common cause of brain tumors in adults, with an incidence of 9-17%, based on various studies, although it was thought to be higher. The aim of this study was to describe recorded cases of metastatic brain tumors in the adult population of a tertiary care and oncology center in Jeddah, Saudi Arabia.

Methods

This study was conducted at King Abdulaziz Medical City (KAMC) at King Khalid Hospital in Jeddah, Saudi Arabia, including records from January 2016 to December 2020. The study implemented a retrospective cohort design to fulfill its aim. A data collection sheet containing demographic data such as age and gender, and information pertaining to the primary pathology, multiplicity, and survival outcome was used.

Results

A total number of 213 patients were enrolled in this study. Overall, 68.1% of the sample comprised of females. Approximately two-thirds (61.9%) of the patients’ imaging results revealed multiplicity, whereas the remaining third (38.1%) had solitary lesions. The estimated overall survival median after the diagnosis of brain metastasis was six months (95% CI: 5.5-6.5).

Conclusion

We recommend conducting a nationwide study to better understand the incidence in accordance to geographical and gender differences. We can further expand our research to include other institutes in Saudi Arabia, and include important predictors such as time from the diagnosis of primary pathology to brain metastasis, disease progression cost, and disease progression in the months prior to the patients’ death.

## Introduction

Although brain tumors account for less than 2% of all tumors worldwide, they are considered one of the most fatal cancers [1]. A total of 251,329 new deaths occurred from a total of 308,102 new cases of brain tumors in 2020, ranking 13th in mortality numbers worldwide [2]. Brain tumors are being increasingly studied in developed countries due to their considerable increase in incidence, poor prognosis, and mortality [1]. In fact, incidence of brain tumors is increasing worldwide. Advanced imaging, higher imaging frequency, and evolution of modern diagnostic techniques are thought to be the cause of this growth [2]. It has been reported that the prevalence of brain tumors in Saudi Arabia’s population is 0.3% [3]. A total of 367 new cases of brain tumors were reported in the latest 2016 report published by the Saudi Cancer Registry, making it the ninth most common cancer in females and the 10th in males in the population of Saudi Arabia [4-5]. Histopathological patterns of brain tumors were not specified in the report, however, and both primary and metastatic brain tumor cases were reported as a whole [6]. As a well-documented fact, metastatic brain tumors are the most common cause of brain tumors in adults with an incidence of 9-17%, based on various studies, although it was thought to be higher [7-8]. Mostly parenchymal, metastases have the ability to spread to various locations of the central nervous system, such as the leptomeninges and dura. The rise in metastatic brain tumors is linked to improved imaging and newer more efficient systemic therapies, which contribute to a higher survival rate and longer life expectancy after diagnosis of the primary cancer [7]. Such advances allow patients to live longer and have higher chances of primary tumor dissemination to the brain [7,9]. Although systemic chemotherapeutic agents control systemic disease, some agents, such as trastuzumab, do not penetrate the blood-brain barrier, failing to protect against brain metastases [7]. In most of the studies published worldwide, lung, breast, melanoma, renal, and colorectal primary cancer patients frequently develop brain metastases [7-8,10]. Nonetheless, racial, geographical, and environmental variation in trends of metastatic brain tumors is evident. For example, metastases from lung and breast cancer in African Americans were higher compared to white Americans in one study [8]. Variation in clinical factors at the time of presentation aids in prediction of the clinical course and the prognosis [9]. The first attempt to describe brain metastases as a part of a larger study in Saudi Arabia describing all intracranial masses (both neoplasms and non-neoplastic) was in 1989 [11]. Available data on brain tumors and brain metastases, in particular, are limited in the Saudi population [7]. In 2011, a single-centered study by Bangash reported and described patterns of metastases in the Saudi population [12]. Therefore, more recent epidemiological studies describing trends are needed to better understand ethnic, geographical, and environmental differences. Conducting such studies will ultimately direct the needed resources to help reach a better outcome. The aim of this study was to describe rates and trends of metastatic brain tumors in the adult population of a tertiary care and oncology center in Jeddah, Saudi Arabia.

## Materials and methods

Study design, area, and settings

This was an observational study conducted at King Abdulaziz Medical City (KAMC) at King Khalid Hospital in Jeddah, Saudi Arabia. The retrospective cohort study design included records from January 2016 to December 2020. KAMC is a non-profit tertiary care hospital in Jeddah, Saudi Arabia, that provides high-standard patient care. Located in KAMC, Princess Noorah Oncology Center, a leading cancer center in the region and the most prominent cancer center in the Western Region of Saudi Arabia, serves the Western, Northern, and Southern parts of the Kingdom.

Identification of study participants

Patients with established primary tumors (controlled or active) outside the central nervous system (CNS) with evidence of brain metastases, aged 18 years and above, and treated in KAMC inpatient and outpatient facilities were included. Those with an initial diagnosis of brain metastasis that was discovered to be a primary CNS pathology, such as primary CNS tumor, abscess, or focus of demyelination, were excluded from the study. To achieve the outcomes of this study, all eligible patients in the five-year study period were entered using non-probability consecutive sampling.

Data collection process

A data collection sheet containing demographic data such as age and gender, and information pertaining to the primary pathology, multiplicity, and survival outcome was used. The authors have collected their data through the National Guard Hospital's BestCare system in Jeddah.

Data analysis

Data recorded were entered and analyzed using Statistical Package for the Social Sciences (SPSS) Version 23 (IBM Corp., Armonk, NY). Categorical data were computed and presented in frequencies and percentages. Continuous data were used based on normality assessments and were presented in means and standard deviations or medians and quartiles to describe the data depending on distribution. Comparison of these variables was done using independent and dependent samples t-test or Mann-Whitney U test for continuous data, as appropriate, and chi-square test for categorical data. P-values < 0.05 were considered significant. A Kaplan-Meier product limit method was used to estimate survival analysis.

## Results

Demographic data

A total number of 213 patients were enrolled in this study. Of the sample, 68.1% comprised of females. The mean age of patients was 53.6 ± 15.9 years. The vast majority of cases had breast cancer as their primary pathology (44.1%), followed by lung cancer (22.1%), renal cancer (6.1%), and colorectal cancer (2.8%). Other types of primary cancers that contributed to brain metastasis totaled to 24.9%. Approximately two-thirds (61.9%) of the patients’ imaging results revealed multiplicity, whereas the remaining third (38.1%) had solitary lesions. In regard to survival outcome, 72.3% of all patients were documented to have passed away. The estimated overall survival median after the diagnosis of brain metastasis was six months (95% CI: 5.5-6.5) (Tables 1, 2).

**Table 1:** Demographic data

Characteristics	N=213
Age (in years)	53.6 ± 15.9
Gender	
Male	68 (31.9)
Female	145 (68.1)
Primary pathology	
Lung cancer	47 (22.1)
Breast cancer	94 (44.1)
Renal cancer	13 (6.1)
Colorectal cancer	6 (2.8)
Others	53 (24.9)
Multiplicity	
Yes	132 (61.9)
No	81 (38.1)
Survival outcome	
Deceased	154 (72.3)
Alive	28 (13.1)
Unknown	31 (14.6)

**Table 2:** Means and medians for survival time M, months

Mean				Median			
Estimate	Std. error	95% confidence interval		Estimate	Std. error	95% confidence interval	
		Lower bound	Upper bound			Lower bound	Upper bound
58.243 M	1.818 M	54.680 M	61.806 M	60.000 M	2.749 M	54.611 M	65.389 M

Analytical analysis

**Figure 1:**
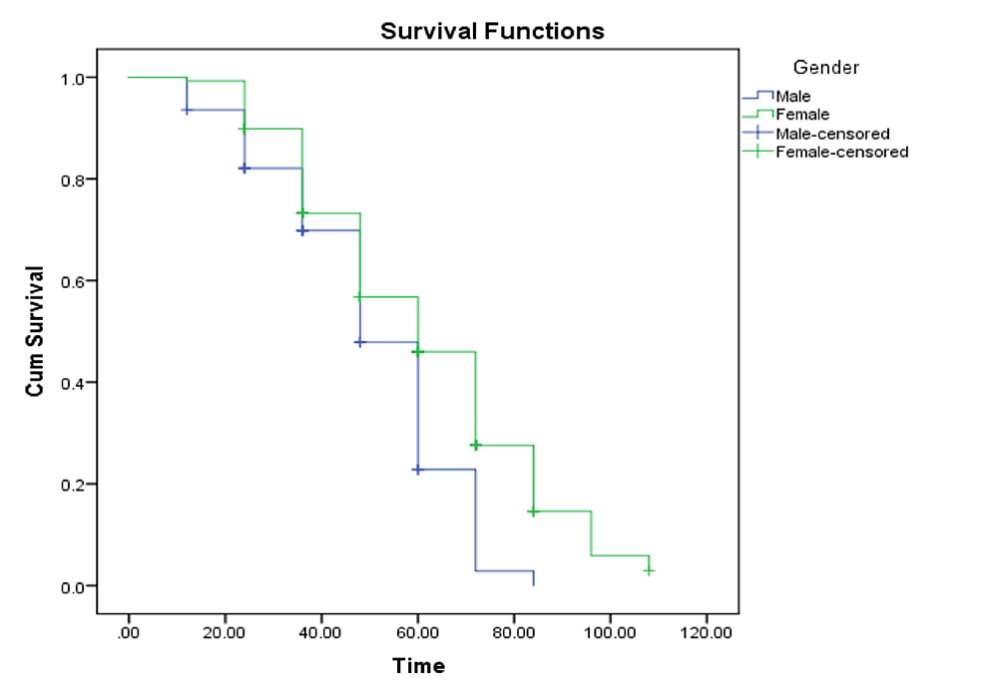
Survival functions based on gender Time in months

There was a significant difference between both genders in terms of survival outcome (p=0.002); 77.4% (48 out of 62) males were announced deceased, while 69.6% of females (96 out of 138) passed away. The survival median for males specifically was 48 months (95% CI: 4.1-5.5), and females’ median equaled 60 months (95% CI: 5.3-6.7). Time of follow-up for 10 deceased patients was missing; therefore, they were not included in Tables 3, 4 and Figure 1.

**Table 3:** Case processing summary in regard to gender

Gender	Total	No. of events	Censored	
			N	Percent
Male	68	54	14	22.6%
Female	145	100	42	30.4%
Overall	213	154	56	28.0%

**Table 4:** Means and medians for survival time based on gender M, months

Gender	Mean				Median				
	Estimate	Std. error	95% confidence interval		Estimate	Std. error	95% confidence interval		
			Lower bound	Upper bound			Lower bound	Upper bound	
Male	50.279 M	2.506 M	45.367 M	55.190 M	48.000 M	3.489 M	41.161 M	54.839 M	
Female	61.578 M	2.281 M	57.107 M	66.049 M	60.000 M	3.724 M	52.702 M	67.298 M	
Overall	58.243 M	1.818 M	54.680 M	61.806 M	60.000 M	2.749 M	54.611 M	65.389 M	

**Figure 2:**
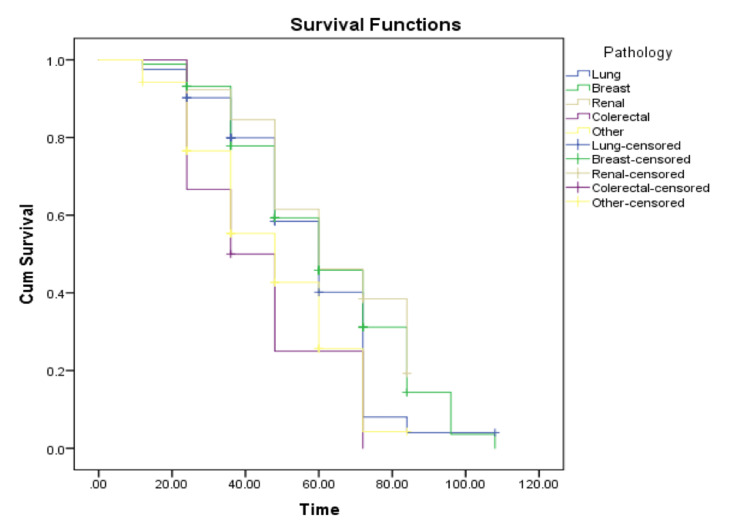
Survival functions based on pathology

In regard to primary pathology, significant differences (p = 0.006) in fatality were shown among the five cancer pathologies listed in Tables 5, 6, and Figure 2. Brain metastasis originating from colorectal cancer resulted in the highest rate of deaths (83.3%), while metastasis rising from primary renal cancer had a rate of 69.2%. Moreover, deaths due to brain metastasis from primary lung cancer and other unspecified primary cancers equaled 70.7 and 73.1, respectively. Even though breast cancer did not result in the highest rate of mortality (71.6%), it did account for the most cases of total deaths secondary to brain metastasis (63 out of 144). Lung, breast, and renal cancers had an estimated median survival of 60 months (95% CI: 4.9-7.1, 5.1-6.9, and 3.2-8.3, respectively). However, colorectal cancer patients had the shortest survival duration of 36 months (95% CI, 1.3-5.3). Finally, other types of metastatic primary malignancies had a survival span of 48 months (95% CI: 3.4-6.2). (Tables 5, 6 and Figure 2).

**Table 5:** Case processing summary in regard to cancer pathology

Pathology	Total	No. of events	Censored	
			N	Percent
Lung	41	29	12	29.3%
Breast	88	63	25	28.4%
Renal	13	9	4	30.8%
Colorectal	6	5	1	16.7%
Other	52	38	14	26.9%
Overall	200	144	56	28.0%

**Table 6:** Means and medians for survival time in regard to pathology M, months

Pathology	Mean				Median			
	Estimate	Std. error	95% confidence interval		Estimate	Std. error	95% confidence interval	
			Lower bound	Upper bound			Lower bound	Upper bound
Lung	57.884 M	3.404 M	51.212 M	64.555 M	60.000 M	5.838 M	48.557 M	71.443 M
Breast	62.902 M	2.690 M	57.629 M	68.175 M	60.000 M	4.782 M	50.628 M	69.372 M
Renal	62.769 M	5.899 M	51.206 M	74.332 M	60.000 M	14.379 M	31.816 M	88.184 M
Colorectal	44.000 M	8.898 M	26.561 M	61.439 M	36.000 M	11.758 M	12.955 M	59.045 M
Other	47.847 M	2.955 M	42.055 M	53.639 M	48.000 M	7.071 M	34.140 M	61.860 M
Overall	58.243 M	1.818 M	54.680 M	61.806 M	60.000 M	2.749 M	54.611 M	65.389 M

**Figure 3:**
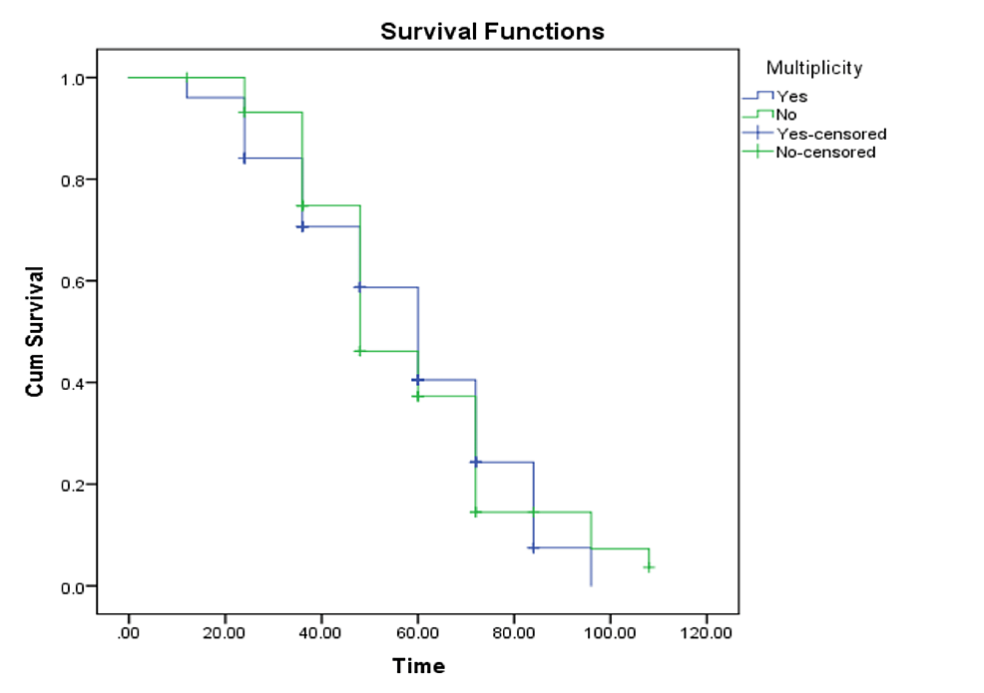
Survival functions based on multiplicity

Brain metastasis characterized by multiplicity was an insignificant predictor of increased mortality with a p-value of 0.89. Overall, 70.6% of patients who had multiple metastatic lesions eventually died, and 74.3% of patients with a single metastatic mass died. Patients with multiple metastatic lesions survived for a median of 60 months (95% CI: 5.4-6.6), whereas patients with solitary lesions lived a median of 48 months (95% CI: 4.0-5.5) (Tables 7, 8 and Figure 3).

**Table 7:** Case processing summary in regard to multiplicity

Multiplicity	Total	No. of Events	Censored	
			N	Percent
Yes	126	89	37	29.4%
No	74	55	19	25.7%
Overall	200	144	56	28.0%

**Table 8:** Means and medians for survival time in regards to multiplicity M, months

Multiplicity	Mean				Median			
	Estimate	Std. error	95% confidence interval		Estimate	Std. error	95% confidence interval	
			Lower bound	Upper bound			Lower bound	Upper bound
Yes	57.822 M	2.221 M	53.468 M	62.176 M	60.000 M	3.132 M	53.860 M	66.140 M
No	58.516 M	2.943 M	52.748 M	64.283 M	48.000 M	3.953 M	40.252 M	55.748 M
Overall	58.243 M	1.818 M	54.680 M	61.806 M	60.000 M	2.749 M	54.611 M	65.389 M

## Discussion

Brain tumors are a diverse group of neoplasm. In the adult population, certain kinds of tumors are more prominent, such as glial neoplasms, meningioma, and metastasis [2]. These tumors are associated with a serious morbidity and high numbers of mortality, regardless of the tumor’s nature or histological grade [2]. Recently, brain metastasis increased as a consequence of the increase of cancer patients’ survival and the increase of use of diagnostic imaging modalities such as MRI [7,10]. The clinical course and prognosis is remarkably different due to certain predetermined factors such as the primary tumor’s histology, number of brain metastases, and extracranial disease state [13].

The results of the current study showed that among the study population, more than half were female patients. In line with prior similar studies, our results show that most cases of brain metastasis patients had their primary pathology as follows: breast cancer (44.1%), lung cancer (22.1%), renal cancer (6.1%), and colorectal cancer (2.8%). Given the fact that more than half of our patients were females, breast cancer was the primary pathology at the top of the list. Rastogi et al. reported that lung (68%) was the most common primary pathology site, followed by breast (11%) [14]. Similarly, Berghoff et al. outlines that breast cancer is the second most common cause of brain metastasis, second to lung cancer [11]. Moreover, Berghoff et al. reported in their study some other tumor types that seldom lead to brain metastasis, such as gastroesophageal cancers, genitourinary cancers, head and neck cancers, and sarcomas [11]. In study results, multiplicity was noted in two-thirds of the patients given their imaging results; hence, one-third of the patients had solitary lesions. Rastogi et al. reported that 80% of their study population had multiple brain lesions [14]. Yang et al. have discussed in their paper that having multiple brain lesions has been considered a negative prognostic factor in patients having brain metastasis [15].

The overall survival of patients following their diagnosis of brain metastasis was six months. In contrast with our results, Rastogi et al also reported a median overall survival rate of six months [14]. However, both Hazuka et al. and Lagerwaard et al. reported different survival rates: 3.4 months and 11 months, respectively [16,17]. A study conducted by Rades et al. showed a median overall survival rate of six months [18]. Although there was a significant difference between survival median for both genders in our study (48 months for males and 60 months for females), Harris et al. reported that gender was not a significant predictor of overall survival [19]. In respect of the survival rates, more than half of the patients enrolled in this study were reported to have passed away; such a result is consistent with that in the study by Bangash, who also reported over half (72.73%) of their participants passing away [13]. However, Rotta et al. reported that only 28% of their patients passed away [20].

The fatality of patients with brain metastases varied significantly according to the primary pathology. Colorectal cancer resulted in the highest death rates, followed by renal cancer, primary lung cancer, and other unspecified primary cancer. Lung, breast, and renal cancers had an estimated median survival of 60 months; however, colorectal cancer had the shortest survival duration (36 months). In contrast, a study conducted by Berghoff et al. reported that patients with breast cancer had the longest median overall survival duration (eight months), followed by patients with lung cancer and renal cell carcinoma (seven months) and melanoma (five months) [11]. Moreover, our study and Berghoff et al.’s study reported different overall median survival duration of colorectal cancer (3.6 months and 40 months, respectively) [10]. Even though Berghoff et al. reported renal cell carcinoma as their second best in terms of survival time, Bangash showed that it had the best overall survival duration [11-13]. Lastly, Tabouret et al. deduced a different but somewhat similar set of overall median survival duration, which is as follows: 2.7-6.3 months for lung cancer, 5.1-6 months for colorectal cancer, and 4.8-10 months for melanoma [21].

While breast cancer does not have the highest rate of mortality among patients with brain metastasis, it is accountable for the most cases of total deaths secondary to brain metastasis. In contrast to our results, Rotta et al. mentioned that lung cancer was responsible for the most deaths [20].

Limitations

Although the research tackles brain metastases and their association to various factors like primary pathology and gender, there are still limitations faced in the study. For example, our study is based on records of a single institute; its results might not reflect the general population despite its concurrence with the existing literature. The study, albeit setting a foundation for future research, has brushed over the topic in a superficial manner and requires extensive research in the future, such as performing comparative studies between the factors discussed in regard to brain metastases.

## Conclusions

Brain metastases are a devastating neurological complication of systemic cancer. It leads to significant morbidity and mortality. The incidence of brain metastasis is increasing with the advancement of diagnostic imaging. Various tumors can metastasize to the brain, our study demonstrated the most prevalent primary pathology tumors to result in a brain metastasis to be breast cancer, lung cancer, renal cancer, and colorectal cancer, respectively. Moreover, multiplicity was noted among patients having brain metastasis. We recommend carrying out a nationwide study to better understand the incidence in accordance to geographical and gender differences. We can further expand our research to include other institutes in Saudi Arabia and include important predictors like time from the diagnosis of primary pathology till brain metastasis, disease progression cost, disease progression in the months prior to the patients’ death.
